# Icatibant in the Treatment of Angiotensin-Converting Enzyme Inhibitor-Induced Angioedema

**DOI:** 10.1155/2014/864815

**Published:** 2014-09-23

**Authors:** Neil H. Crooks, Jaimin Patel, Lavanya Diwakar, Fang Gao Smith

**Affiliations:** ^1^Academic Department of Anaesthesia, Critical Care, Pain and Resuscitation, Heart of England NHS Foundation Trust, Birmingham Heartlands Hospital, 1st Floor MIDRU Building, Bordesley Green East, Birmingham B9 5SS, UK; ^2^Department of Infection and Immunity, Queen Elizabeth Hospital, Birmingham B15 2TH, UK; ^3^Perioperative, Critical Care and Trauma Trials Group, School of Clinical and Experimental Medicine, University of Birmingham, Birmingham B15 2TT, UK

## Abstract

We describe the case of a 75-year-old woman who presented with massive tongue and lip swelling secondary to angiotensin-converting enzyme inhibitor-induced angioedema. An awake fibre-optic intubation was performed because of impending airway obstruction. As there was no improvement in symptoms after 72 hours, the selective bradykinin B2 receptor antagonist icatibant (Firazyr) was administered and the patient's trachea was successfully extubated 36 hours later. To our knowledge this is the first documented case of icatibant being used for the treatment of angiotensin-converting enzyme inhibitor-induced angioedema in the United Kingdom and represents a novel therapeutic option in its management.

## 1. Introduction

Angiotensin-converting enzyme inhibitor- (ACEi-) induced angioedema is one form of bradykinin-mediated angioedema. It occurs in less than 1% of patients receiving treatment with ACEi. The effect is not dose-related and is a class effect. It may occur at any time after beginning treatment with the drug, with 60% of patients developing symptoms within the first week [1]. The tongue, lips, and eyes are predominantly affected with stridor or respiratory failure occurring in 11% of cases [2].

Icatibant, a highly selective bradykinin B2 receptor antagonist, is used in the treatment of acute attacks of hereditary angioedema [3]. However, several case reports have documented that it can be used safely and successfully for the treatment of ACEi-induced angioedema. We describe what we believe to be the first reported case in the UK of ACEi-induced angioedema treated successfully with icatibant.

## 2. Case Report

A 75-year-old hypertensive, diabetic, Afro-Caribbean lady presented to the emergency department with a two-hour history of progressive tongue swelling. On arrival she was unable to speak or swallow secretions but had no evidence of stridor. She had profound tongue and lip swelling consistent with angioedema. On further examination, there was no evidence of rash or wheeze. She had no known allergies.

Ramipril (2.5 mg OD) had been prescribed 3 days earlier by her general practitioner in addition to her other regular medication, atenolol, amlodipine, bendroflumethiazide, and metformin. She had so far taken two 2.5 mg tablets of ramipril, the last one on the preceding evening.

Initial observations showed an arterial oxygen saturation of 99% on room air with no evidence of cardiovascular compromise. Immediate management involved delivery of high flow oxygen, intramuscular adrenaline 0.5 mg, intravenous hydrocortisone 200 mg, and intravenous chlorphenamine 10 mg. Despite these measures the tongue swelling continued and, in view of the onset of stridor, the patient was transferred to theatre for an awake fibre-optic intubation. This was performed with an ENT surgeon on stand-by and proved extremely difficult due to severe distortion of the upper airway anatomy.

The patient was subsequently transferred to the Intensive Care Unit (ICU) for supportive management. After 72 hours there had been no resolution of symptoms despite regular administration of dexamethasone and chlorphenamine. After discussion with the immunology team it was felt appropriate to consider the off-license use of icatibant (Shire Human Genetic Therapies Inc.) for this patient, as the risks of continued intubation and ventilation were considered greater than the use of icatibant.

**Figure 1:**
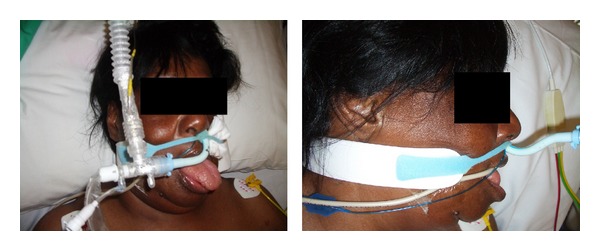
Before and after treatment with icatibant.

Icatibant is administered as a 30 mg subcutaneous injection into the anterior abdominal wall, up to a maximum dose of 90 mg within 24 hours. The first dose was administered on ICU day four and a significant reduction in tongue swelling was noticed within 30 minutes (Figure 1). Two further doses were administered 8 hours apart by which time the tongue swelling had almost completely resolved.

**Figure 2:**
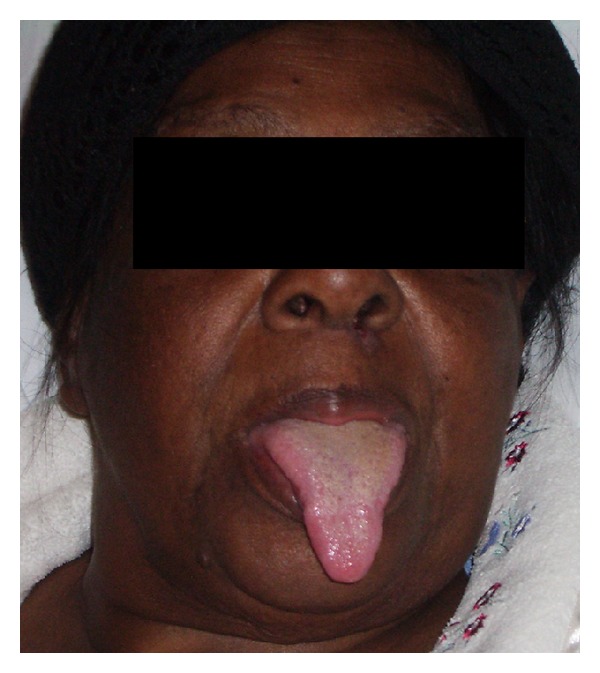
After extubation.

Despite resolution of angioedema within the first few hours of icatibant administration, weaning from mechanical ventilation was delayed as the patient had developed a ventilator-associated pneumonia. Successful extubation eventually took place 36 hours after the first dose of icatibant. No adverse effects were noted apart from mild erythema around the injection site. The patient was discharged to the ward on day six (Figure 2), where she required a period of rehabilitation, after which she was discharged home. She was advised to avoid ACEi in the future.

## 3. Discussion

Angiotensin-converting enzyme inhibitors (ACEi) are one of the most commonly prescribed antihypertensive drugs and are a well-known, but often unrecognised, cause of angioedema. The incidence of ACEi-induced angioedema is between 0.1%–0.7% which may progress to airway compromise [1, 4].

**Figure 3:**
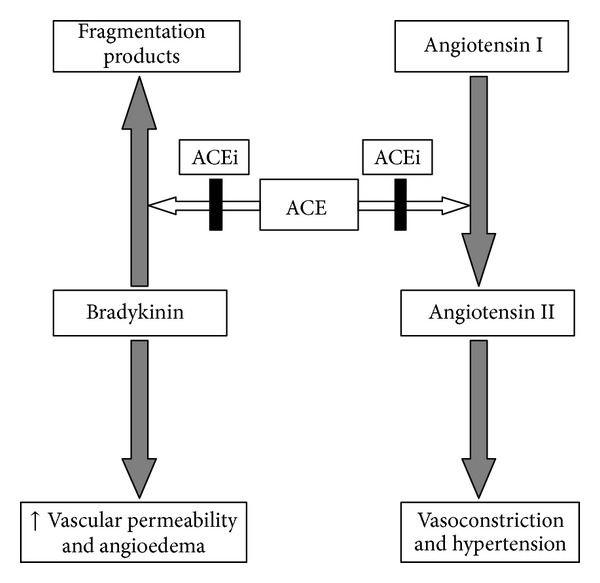
Mechanism of ACEi-induced angioedema.

ACEi block the conversion of angiotensin-I to angiotensin-II, lowering arteriolar resistance and increasing venous capacitance, and thereby exerting an antihypertensive effect. ACEi also inhibit the breakdown of bradykinin into its fragmentation products leading to an accumulation of circulating bradykinin. It is this accumulation which leads to one of the commonest side-effects of ACEi, the dry cough. In some patients however, the accumulation of bradykinin also leads to increased vascular permeability, interstitial oedema, and finally angioedema (Figure 3).

This is very similar to the pathophysiology of hereditary angioedema which is mediated mainly by bradykinin-induced activation of vascular bradykinin B2 receptors [4]. This explains why traditional therapies for “allergic” angioedema (e.g., food allergy) are ineffective, as none act directly or indirectly on the bradykinin pathways responsible for the angioedema [4, 5].

Icatibant is a synthetic decapeptide which competitively inhibits bradykinin B2 receptors on the vascular endothelium. It displaces endogenous bradykinin and leads to reversal of angioedema by reducing vascular endothelial permeability.

Icatibant was licensed in 2008 for use in hereditary angioedema [6, 7]. A phase III randomised controlled trial comparing icatibant to placebo or tranexamic acid demonstrated a significant reduction in time to subjective and objective resolution of symptoms in the icatibant group [3]. Interest in icatibant for the treatment of ACEi-induced angioedema is growing with a recent case series showing significant symptom reduction within a mean time of 50.6 minutes [4]. In this case series none of the patients required tracheal intubation or tracheostomy tube insertion and no serious adverse events were reported.

Icatibant has a high bioavailability (97%) and maximal concentration in the serum is achieved in around 30 minutes [7]. A single dose of the drug has been shown to be effective in the majority of cases [8]. However, the terminal plasma half-life of icatibant is 1-2 hours and hence more than one dose may be necessary. Local reactions (pain, erythema, and swelling) are common side-effects and the drug is not recommended in individuals with acute cardiac or brain ischaemia [6, 7].

Due to the acute presentation of ACEi-induced angioedema and its low incidence, the use of icatibant in this situation has previously been off-license and confined to case reports and case series. However, a phase III, randomized, controlled trial evaluating the safety and efficacy of icatibant as a treatment for ACEi-induced angioedema in adults is currently ongoing and we await the results of this trial with interest [9].

## 4. Conclusion

The increasing prevalence of ACEi use indicates that the frequency of related cases of angioedema will also increase. The use of icatibant is well-recognised among the immunology community, but knowledge of its benefits is extremely limited in the acute care setting. Increasing awareness of this drug's existence as a potential alternative to intubation and ventilation may lead physicians to consider it as a treatment option early in the course of the disease.
